# Application of image analysis in the evaluation of blood feeding by *Lutzomyia longipalpis* using an artificial feeding system with synthetic membranes

**DOI:** 10.1590/0074-02760260065

**Published:** 2026-10-16

**Authors:** Lidiane Medeiros da Costa, Maurício Roberto Viana Sant’Anna, Laura Aquino Vaz, Geisler Peixoto da Cruz, Luisa de Melo Lara, Gabriel Davi Medeiros de Paula, Ricardo Nascimento Araujo, Nelder de Figueiredo Gontijo, Marcos Horacio Pereira

**Affiliations:** 1 Universidade Federal de Minas Gerais, Instituto de Ciências Biológicas, Departamento de Parasitologia, Laboratório de Fisiologia de Insetos Hematófagos, Belo Horizonte, MG, Brasil; 2 Pontifícia Universidade Católica de Minas Gerais, Belo Horizonte, MG, Brasil; 3 Universidade Federal de Minas Gerais, Instituto de Ciências Biológicas, Departamento de Parasitologia, Laboratório de Artrópodes Hematófagos, Belo Horizonte, MG, Brasil

**Keywords:** Lutzomyia longipalpis, artificial feed, collagen membrane, blood feed, plasma gel, image analysis

## Abstract

**BACKGROUND:**

Blood feeding is essential for the maintenance of laboratory colonies and for experimental studies involving sand fly-parasite interactions. However, artificial feeding systems for *Lutzomyia longipalpis*, the principal vector of *Leishmania infantum*, the etiological agent of visceral leishmaniasis in the Americas, remain limited by low engorgement rates and methodological constraints in blood meal quantification.

**OBJECTIVES:**

This study evaluated the feeding performance of *Lu. longipalpis* on different membranes in an artificial feeder and applied an image-based approach to quantify blood meal size.

**METHODS:**

Females were fed either on anaesthetised hamsters (*in vivo* control) or using a Hemotek^®^ artificial feeder equipped with chick skin (CHK), hydrated collagen membrane treated with plasma gel (COL+PG), or untreated hydrated collagen membrane (COL). Blood meal size was estimated through digital image analysis in Fiji/ImageJ using two regions of interest: whole abdomen area (AbdA) and red-coloured blood area (BldA).

**FINDINGS:**

Feeding rates were highest on live hosts (88.75%), followed by CHK (72.34%) and COL+PG (72.08%), whereas COL yielded significantly lower engorgement (54.67%). Although abdominal area was greater in females fed on hamsters, no significant differences in red-coloured area were detected among artificial membrane treatments. A strong positive correlation was observed between AbdA and BldA measurements (R² = 0.85), validating the image-based method. The use of plasma-treated collagen membranes achieved feeding rates comparable to CHK and was fully compatible with semi-automated image analysis.

**MAIN CONCLUSIONS:**

These findings demonstrate that hydrated collagen membranes treated with plasma gel constitute a viable and ethically preferable alternative to bird-derived membranes for the artificial feeding of *Lu. longipalpis*, while image analysis provides a precise, reproducible, and practical method for blood meal quantification. Collectively, this approach refines laboratory experiments *Lu. longipalpis* and enhances methodological reliability in studies of sand fly biology and vector-parasite interactions.

Currently, 1063 sand fly species are described, of which 82 species belong to *Lutzomyia*, including more than 40 proven *Leishmania* vectors.^1^ ^,^ ^2^ Some species also transmit bacteria, arboviruses, and can cause allergic reactions such as Harara, a severe chronic papular urticarial reaction induced by sand fly bites.^3^ *Lutzomyia longipalpis* is the principal vector of *Leishmania infantum*, the etiological agent of visceral leishmaniasis in the Americas, transmitting *L. infantum* between dogs and humans.^4^ ^,^ ^5^ Considered a cryptic complex, it shows genetic, pheromonal, and behavioural divergence.^6^ ^,^ ^7^ It is widely distributed from Mexico to northern Argentina and Paraguay, and across most of Brazil, including recent records from the western Amazon.^8^ ^,^ ^9^ ^,^ ^10^ Its ability to colonise both natural habitats (tree crevices, burrows, nests) and peridomestic sites (chicken coops, kennels, pigsties, cattle sheds, organic debris) underlies its epidemiological significance.^7^ ^,^ ^11^ ^,^ ^12^ ^,^ ^13^ ^,^ ^14^

Blood feeding is a critical event for both colony maintenance and experimental studies on parasite transmission. Traditionally, blood meals are obtained from live hosts, which provide chemical, thermal, and behavioural cues that promote high engorgement rates.^15^ However, the use of live animals poses ethical and logistical constraints, prompting the development of alternative methods based on artificial feeding systems.^16^ In this context, the choice of membrane is a key determinant of feeding performance, influencing not only engorgement rates but also subsequent oviposition and progeny viability.^17^

Among animal-derived membranes, chicken skin is widely regarded as the gold standard for several sand fly species, including *Lu. longipalpis*, providing consistently high feeding rates under experimental conditions.^16^ ^,^ ^18^ ^,^ ^19^ More recently, duck foot interdigital membranes have shown comparable performance, representing a viable alternative that reduces the need for neonatal chicks.^20^ Pig intestine, by contrast, has yielded inconsistent and often poor results, particularly for *Lu. Longipalpis*.^20^

Artificial membranes such as collagen or silicone generally produce markedly lower feeding rates compared with biological membranes. In trials with *Lu. longipalpis*, engorgement rates are often close to zero, limiting their use for colony maintenance.^20^ For species such as *Lu. migonei* and *Phlebotomus duboscqi*, performance is moderate but still insufficient to ensure stable colony production.^20^ Even after experimental adjustments, such as the application of coagulated plasma to the membrane surface, feeding rates rarely approach those obtained with animal-derived membranes.^20^ ^,^ ^21^

Comparisons of *in vivo* and *in vitro* feeding further underscore the importance of membrane selection. While live hosts such as hamsters and mice yield high engorgement rates,^15^ ^,^ ^22^ artificial feeders can only achieve satisfactory results when combined with highly accepted membranes such as chicken skin or duck foot, biological membranes generally outperform synthetic alternatives, limiting the widespread application of the latter.^17^ ^,^ ^20^

Understanding how feeding performance varies among live hosts, artificial feeders, and different membrane types is essential for improving sand fly rearing protocols, optimising experimental design, and reducing reliance on live animals. In this context, the present study systematically evaluates the performance of different membranes for the artificial feeding of *Lu. Longipalpis*. By incorporating image analysis techniques to estimate blood meal size, we introduce an accurate, non-invasive, and reproducible approach that improves experimental reliability and opens new avenues for investigating host-parasite-vector interactions.

## MATERIALS AND METHODS

*Insects* - *Lu. longipalpis* originating from Teresina (Piauí, Brazil) were used in this study. The sand flies have been maintained at the insectary of the Laboratory of Haematophagous Insect Physiology (ICB-UFMG) since 2009, under controlled temperature (25 ± 1ºC) and relative humidity (80-95%). Rearing followed the methodology of Modi and Tesh.^3^ After emergence, adults were transferred to nylon rearing cages enclosed within plastic bags containing a sponge soaked in water to maintain high humidity (> 80%). Insects were provided ad libitum with 15% sucrose solution on cotton pads and maintained under a 12:12 h photophase - scotophase photoperiod. Blood feeding was conducted weekly on hamsters (*Mesocricetus auratus*) aged four-eight weeks and weighing 100-150 g. The animals were anesthetised intraperitoneally with sodium thiopentax (Thiopentax^®^, Cristália, Brazil), prepared at a concentration of 50 mg/mL, and administered at a dose of 100 μL per 100 g of body weight (50 mg/kg). All procedures complied with the standards established by the Ethics Committee on the Use of Animals of the Federal University of Minas Gerais (protocol CEUA 262/2021).

*Feeding assays* - A total of 1300 female and 217 male sand flies, five-seven days post-emergence, were used. Insects were provided with 15% sucrose solution *ad libitum* until 12 h before the experiments. Five experimental groups were tested: a group fed on a live host, three on an artificial feeder, and one unfed (negative control, n = 240). Each experiment was performed in four biological replicates.

*Live host* - Golden hamsters (*M. auratus*; 40-60 days old, 100-150 g) were obtained from the animal facility of the Department of Parasitology (ICB-UFMG) and maintained with food and water *ad libitum*. Animals had no previous exposure to sand flies. One group of sand flies was fed on the shaved abdominal skin of anaesthetised hamsters (IVH group, n = 240), which served as a positive control since feeding success is generally higher on live hosts.

*Artificial feeder* - For artificial feeding assays, insects were placed in four labelled pots containing ~60 females and ~10 males each. Males were included to stimulate female feeding behaviour.^23^ Heparinised human blood (0.04 µL heparin/mL blood) collected by venipuncture into Vacutainer^®^ tubes was used both for feeding and plasma gel preparation. Plasma gel was applied to the collagen membrane to provide an olfactory stimulus and enhance feeding attraction. Blood meals were offered using a Hemotek system (Hemotek Ltd., UK) at 37ºC for 90 min. Human blood usage followed the guidelines of the Research Ethics Committee of the Federal University of Minas Gerais (CEP/UFMG protocol 4.512.912).

For blood meals assays in artificial feeder were used three feeding membranes: (i) chick skin (CHK) (positive control; CHK group, n = 235);^19^ (ii) hydrated collagen membrane (Hemotek Feeding Membrane, Hemotek Ltd., UK) treated with plasma gel (COL+PG group, n = 240); and (iii) untreated hydrated collagen membrane (COL group, n = 225). Blood in the Hemotek reservoir was carefully homogenised halfway through the feeding period (~45 min). All experiments were performed under controlled insectary conditions (25 ± 1ºC; 80-95% RH).

*Membrane treatments and plasma gel preparation* - CHK was obtained from birds younger than 10 days, stored at -20ºC in aluminium foil inside sealed plastic bags to prevent desiccation, and thawed to room temperature before use. Collagen membranes (5 × 5 cm) purchased from the Hemotek^®^ website (www.hemotek.co.uk). Plasma gel was prepared from 4 mL heparinised blood (0.2 µL/mL), centrifuged at 4000 g for 15 min to separate plasma. The plasma was transferred to a Petri dish and subjected to 6-20 microwave heating cycles (2 s each, maximum power). Consistency was checked every two cycles, and heating stopped once a fully gelatinous appearance was achieved. Plasma gel was then applied onto hydrated collagen membranes (Fig. 1).

**Fig. 1:**
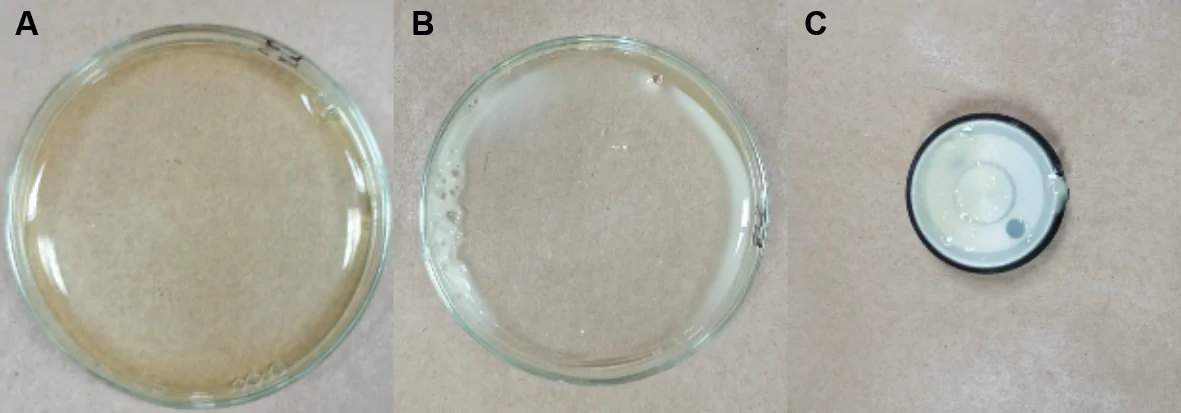
representative image of plasma gel membrane treatment. (A) Plasma obtained after blood centrifugation; (B) Plasma gel formed after heating cycles, exhibiting its characteristic gelatinous appearance; (C) Artificial feeder reservoirs fitted with collagen membranes treated with plasma gel.

In preliminary tests, the collagen membrane was either used dry or hydrated in sterile 0.9% saline for 20 min before attachment (Fig. 2). In these tests, it was observed that the females of *Lu. longipalpis* were not interested in the dry membrane, which was significantly different from what was observed with the hydrated membrane (t-test p < 0.05). Thus, in present study was used only hydrated collagen membrane.

**Fig. 2:**
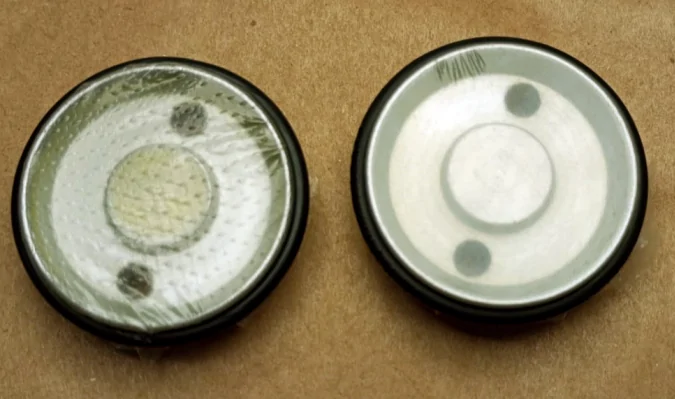
representative image of plasma gel membrane treatment. (A) Plasma obtained after blood centrifugation; (B) Plasma gel formed after heating cycles, exhibiting its characteristic gelatinous appearance; (C) Artificial feeder reservoirs fitted with collagen membranes treated with plasma gel. Artificial feeder (Hemotek^®^) reservoirs with collagen membranes. On the left, a non-hydrated membrane; on the right, a membrane hydrated with 0.9% NaCl.

*Image acquisition* - Immediately after feeding assays, insects were euthanised by freezing at -20ºC for 10 min. Females were grouped in sets of five and imaged using a Canon EOS 6D camera mounted on an Olympus SZ61 stereomicroscope (2 × magnification, base illumination). Images were captured in RGB format at 1920 × 1080-pixel resolution.

*Image processing and analysis* - Images were processed using Fiji/ImageJ (version 1.53f51; https://imagej.net/). Spatial calibration was performed using the Histogram and Set Scale functions. Brightness and contrast were adjusted when necessary to optimise segmentation while preserving the original pixel information. Blood meal size was estimated following the image analysis protocol described by Costa et al.,^24^ with minor modifications. From Red, Green, Blue (RGB) images of the entire insect, two regions of interest (ROIs) were defined: (i) the whole abdomen (AbdA) and (ii) the region corresponding to the blood-containing area (BldA).

For AbdA determination, the abdomen was first separated from the thorax using the polygon selection tool followed by the Clear Outside command. Bristles were reduced by median filtering to minimise segmentation artefacts. The abdominal contour was then delineated after conversion to eight-bit grayscale, threshold adjustment, and automatic boundary detection using the Wand (tracing) tool (Fig. 3A-B). For BldA extraction, the Replace Red with Magenta tool was applied to enhance contrast between blood and surrounding tissues. RGB images were subsequently converted to CMYK colour space, and the magenta channel (32-bit) was isolated. Thresholding was then applied to delineate the blood-filled area (Fig. 3C-E). ROI areas were quantified using the Area measurement option within the Set Measurements panel. A custom ImageJ macro was developed to semi-automate BldA ROI extraction and area quantification, thereby streamlining the workflow and enhancing reproducibility of image-derived measurements (see Supplementary data).

**Fig. 3:**
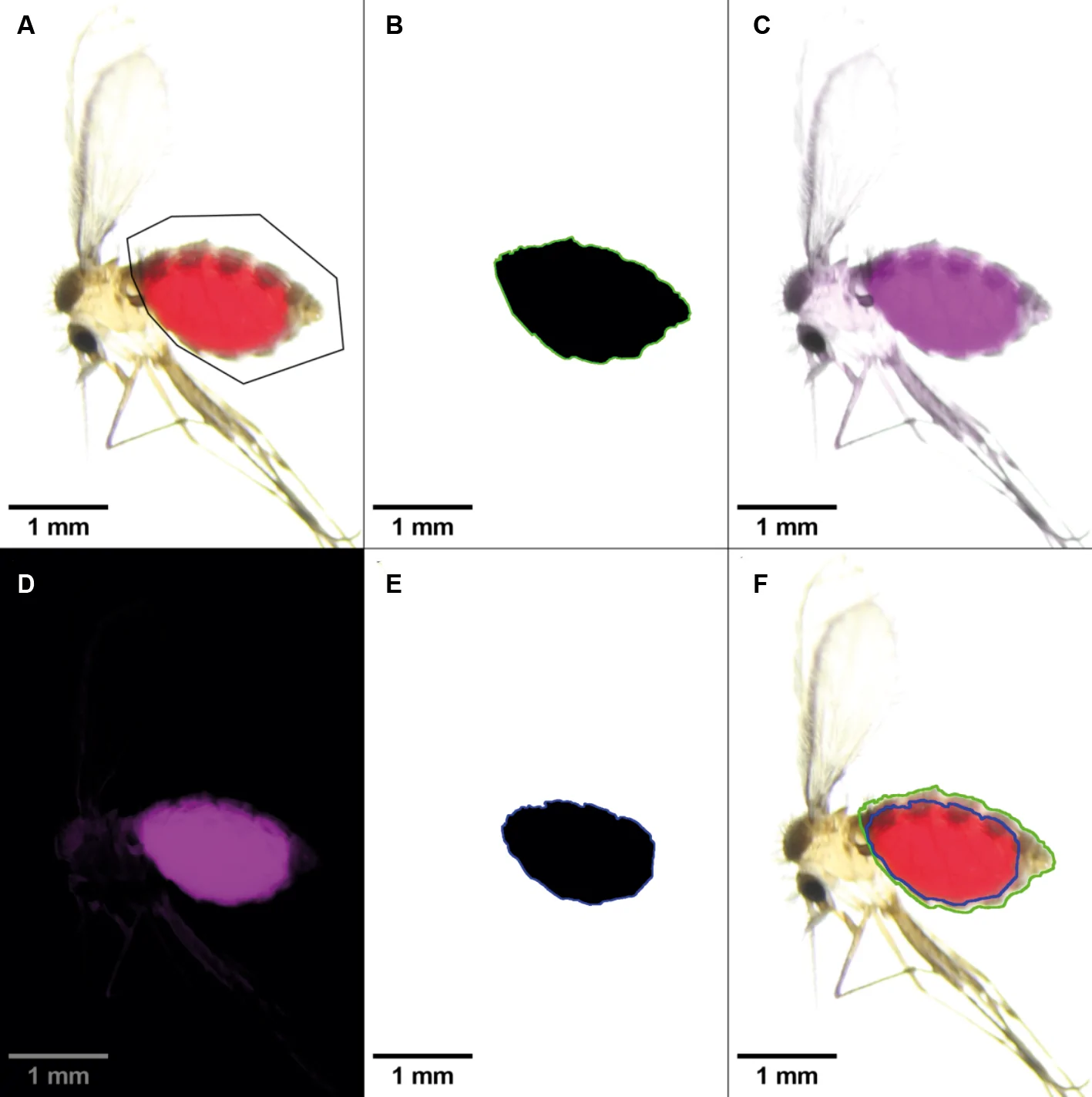
representative image of the flowchart for defining regions of interest (ROIs) in the body of *Lutzomyia longipalpis*. (A-B) Selection of the abdominal contour (AbdA). (C-E) Selection of the contour of the blood-filled region (BldA) in engorged females. (F) Overlay of selections, green line: AbdA contour. Blue line: BldA contour.

*Statistical analysis* - Statistical analyses were performed using GraphPad Prism version 10.1 (GraphPad Software, USA). Data normality was assessed using the Kolmogorov-Smirnov test. For variables exhibiting normal distribution, comparisons between two groups were conducted using the unpaired Student’s t-test, whereas comparisons among more than two groups were performed using one-way analysis of variance (ANOVA) followed by Tukey’s multiple comparisons post hoc test. For non-normally distributed variables, comparisons between two groups were performed using the Mann-Whitney U test. Differences in proportions were analysed using the chi-square test. Data are presented as mean ± standard deviation (SD), and statistical significance was established at p < 0.05. The strength and direction of the association between image-derived areas (AbdA and BldA) were evaluated using Pearson’s correlation coefficient.

## RESULTS

*Proportion of engorged females under different experimental treatments* - The initial evaluation of females after feeding experiments was based on visual inspection of the presence of blood within the abdomen. Females in which blood was clearly detectable in the images were classified as engorged. To improve detection of small blood volumes, particularly in females that had ingested minimal amounts, the pseudo flat-field tool was employed. However, in four assays on CHK membranes, a small proportion of females fed exhibited abdominal distension with a pale appearance, suggesting ingestion of fluid or secretion with little or no erythrocyte content (Fig. 4).

**Fig. 4:**
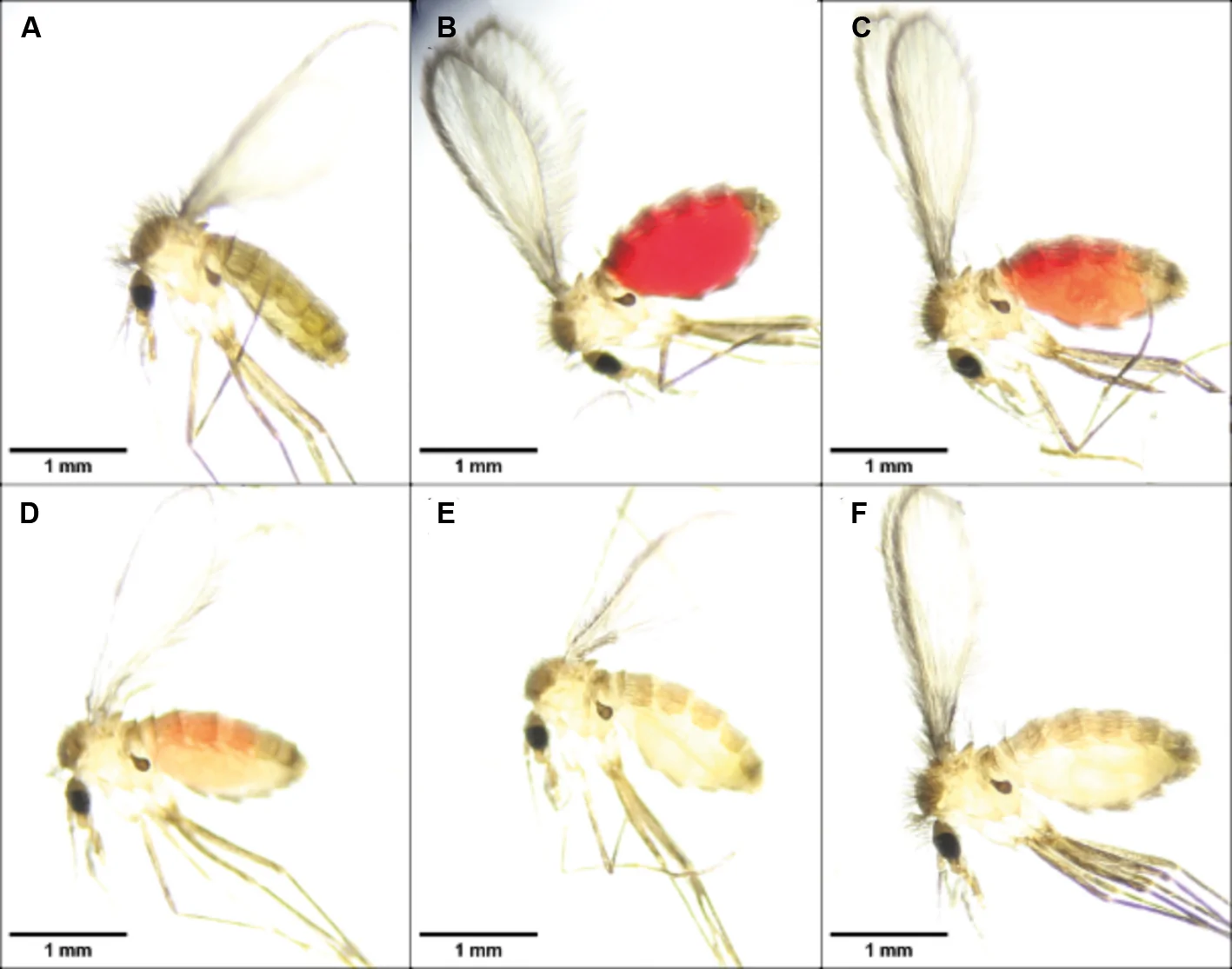
representative image of female *Lutzomyia longipalpis* before (A) and after feeding on blood in an artificial feeder with a chick skin (CHK) membrane, showing abdominal distension with typical (B) or atypical (C-F) colour pattern.

A subset of females from the CHK membrane group (17 females) exhibited atypical abdominal coloration. Because visual assessment alone could not reliably confirm blood ingestion, females with abdominal areas exceeding the unfed group (negative control, n = 240) mean plus two standard deviations (0.62 mm) were considered engorged. According to this criterion, 17 of the 235 females (7.2%) were reclassified as engorged, despite their pale abdominal content.

The proportion of females that successfully fed under the different experimental conditions is shown in Fig. 5. Females fed on hamsters (IVH) exhibited the highest feeding rate (88.75%), confirming the preference of *Lu. longipalpis* for live hosts. Feeding success on CHK and on hydrated collagen membranes treated with plasma gel (COL+PG) was comparable (72.34% and 72.08%, respectively), indicating that both represent viable alternatives for the artificial feeding of *Lu. longipalpis*. In contrast, untreated collagen membranes (COL) yielded the lowest feeding rate (54.67%), significantly lower than the other groups (p < 0.05), although still allowing more than half of the females to engorge (Fig. 5).

**Fig. 5:**
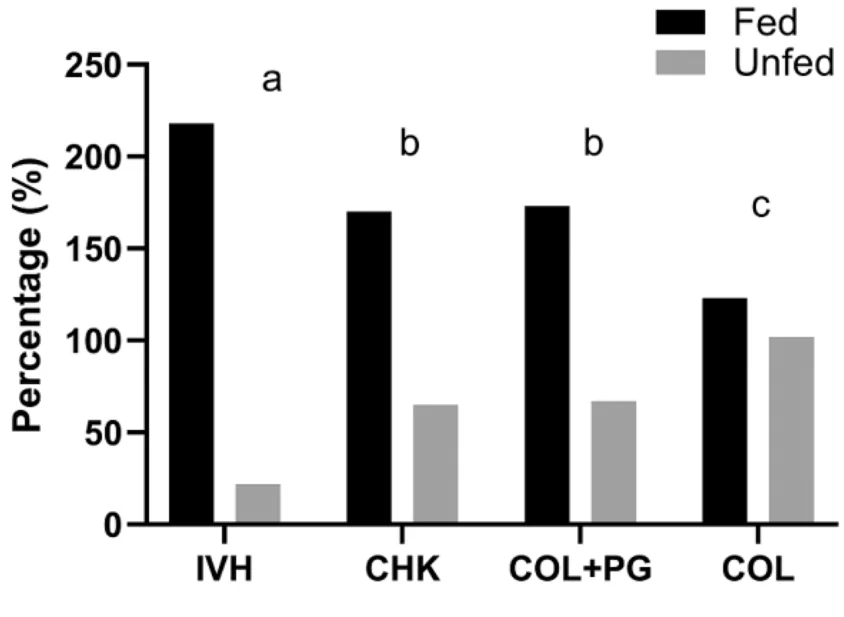
percentage of *Lutzomyia longipalpis* females that successfully fed in the different experimental groups: hamster (IVH), chick skin (CHK), hydrated collagen membrane treated with plasma gel (COL+PG), and untreated hydrated collagen membrane (COL). Different letters indicate statistically significant differences between groups (chi-square test, p < 0.05). Sample sizes were IVH (n = 240), CHK (n = 235), COL+PG (n = 240), and COL (n = 225).

*Quantification of blood meals by image analysis (ROIs AbdA and BldA)* - For the analysis of abdominal area (ROI AbdA), all *Lu. longipalpis* females classified as engorged (see above) were included. The IVH group displayed the largest mean abdominal area (1.20 mm²), which was significantly greater than that observed in the CHK (1.08 mm²) and COL+PG (1.12 mm²) groups (p < 0.0003). No significant difference was detected between CHK and COL+PG. The COL group (1.13 mm²) differed significantly only from CHK (p < 0.0262) (Fig. 6A).

The methodology for selecting the red area (ROI BldA) excluded *Lu. longipalpis* females that ingested fluids lacking the characteristic colouration of blood. Analysis of the blood-filled abdominal area revealed that females in the IVH (0.94 mm^2^) group achieved the highest feeding efficiency, consistent with the results obtained from the AbdA evaluation, thereby reaffirming the preference of females for *in vivo* feeding. In contrast, no statistically significant differences were detected among the CHK (0.85 mm^2^), COL+PG (0.84 mm^2^), and COL (0.84 mm^2^) groups, indicating comparable feeding performance across these artificial membrane treatments (Fig. 6B).

**Fig. 6:**
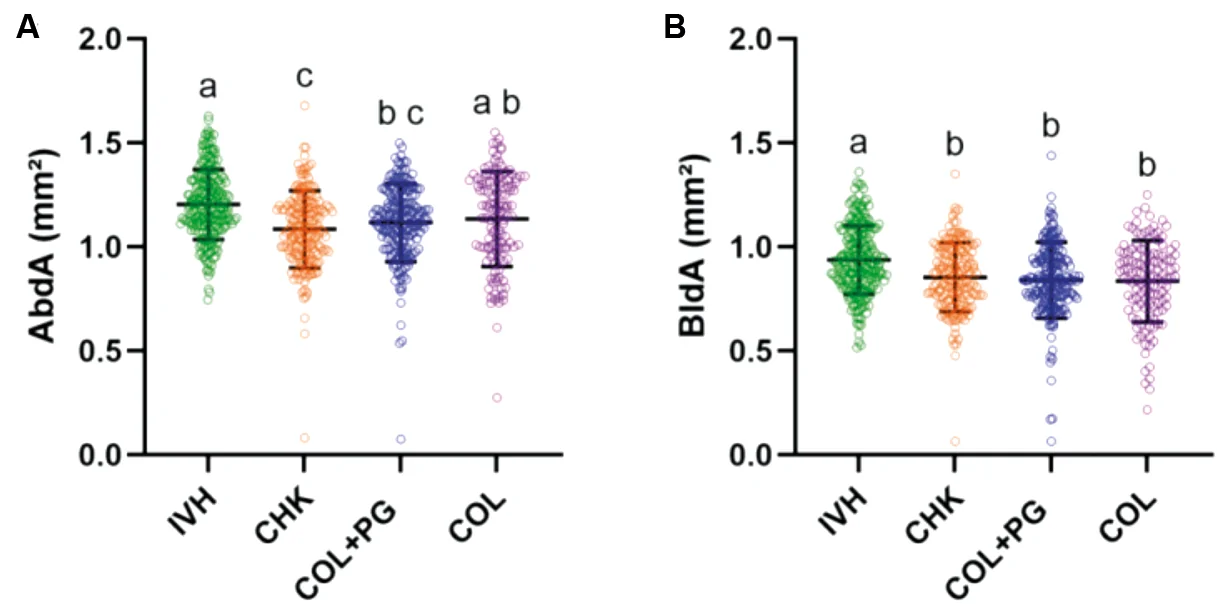
estimation of blood meal size in *Lutzomyia longipalpis* females fed on a live host or on artificial feeders with different membranes. (A) Abdominal area (ROI ABS) and (B) red-coloured region area (ROI BldA), measured in mm². Groups: IVH - hamsters (n = 218), CHK - chick skin (n = 170 for AbdA; n = 155 for BldA), COL+PG - collagen membrane with plasma gel (n = 173), and COL - untreated collagen membrane (n = 123). Different letters indicate statistically significant differences between groups (Kruskal-Wallis, p < 0.05).

When comparing blood meal size estimates derived from the two masks (AbdA and BldA, n = 669), results were highly consistent across experimental groups (Fig. 7). Linear correlation analysis of females with both ROIs measured demonstrated a strong positive association between abdominal area and red-coloured region area (R² = 0.85) (Fig. 7).

**Fig. 7:**
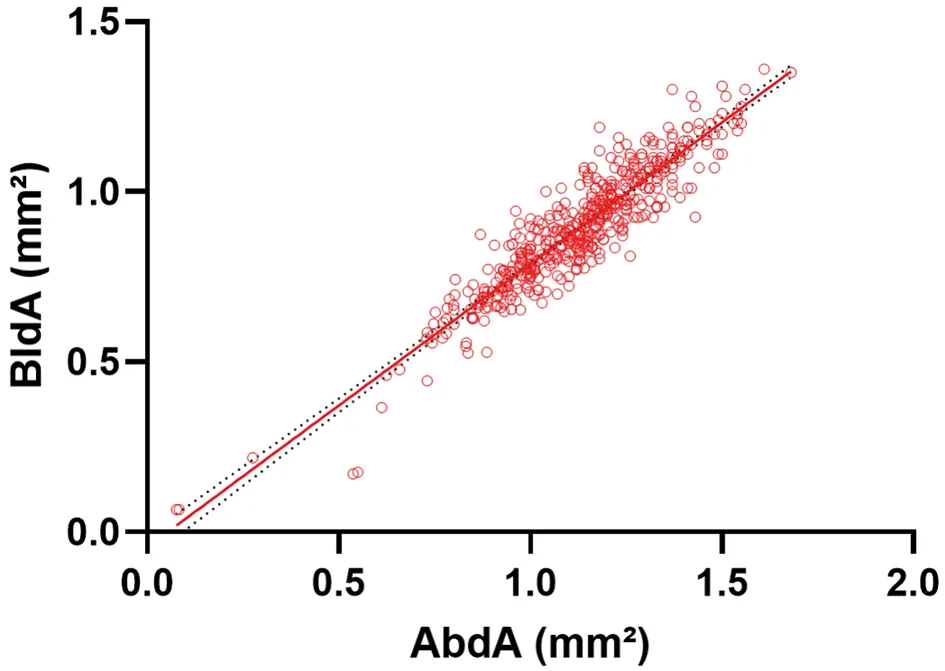
linear regression between abdominal region area (AbdA) and blood-fed region area (BldA) derived from image analysis of 669 fed *Lutzomyia longipalpis* females. The line equation is Y = 0.8320*X - 0.04417, with R² = 0.8531 and p < 0.0001. Dashed lines indicate the 95% confidence interval (0.8008 to 0.8633).

*Comparative feeding performance across experimental groups* - The comparison of feeding performance parameters of *Lu. longipalpis* among the different experimental groups is summarised in Table. Females fed on hamsters consistently exhibited the highest performance across all metrics, reaffirming their preference for live hosts.

**TABLE:** Comparison of feeding parameters in *Lutzomyia longipalpis* females fed on a live host, chick skin (CHK) membrane, and hydrated collagen membrane (with or without plasma gel)

Experimental group	Females fed (%)	Average an abdominal area (mm²)	Average blood area (mm²)	Performance interpretation
IVH (live hamster)	88.75	1.20	0.94	Better performance: higher feeding rate and greater bloodmeal size.
Chick skin (CHK)	72.34	1.08	0.85	Performance similar to COL+PG, some females ingested non-blood fluids.
COL+PG (collagen membrane + plasma gel)	72.08	1.12	0.84	Performance similar to CHK, proving to be a viable alternative to the use of chick skin.
COL (untreated collagen membrane)	54.67	1.13	0.84	Lower % of females fed, but volume ingested similar to CHK and COL.

Among the groups fed using artificial feeders, females provided with CHK membranes, hydrated collagen membranes treated with plasma gel (COL+PG), or hydrated collagen membranes without plasma gel (COL) displayed similar blood meal sizes, with mean abdominal areas (AbdA) of approximately 1.1 mm² and red-coloured region areas (BldA) of 0.84-0.85 mm². However, feeding rates differed between treatments: while CHK and COL+PG groups achieved comparable engorgement proportions (72.34% and 72.08% respectively), the COL group showed the lowest feeding rate (54.67%).

## DISCUSSION

The feeding performance of *Lu. longipalpis* on artificial feeders is notably limited compared with other sand fly species and haematophagous arthropods. High engorgement rates occur only on avian-derived membranes, such as chicken skin — long recognised as the gold standard since early studies^16^ ^,^ ^18^ ^,^ ^19^ — and on duck foot webbing, which shows comparable efficiency.^20^ These laboratory observations are consistent with field evidence indicating the attraction of *Lu. longipalpis* to chickens and other birds.^25^ ^,^ ^26^ ^,^ ^27^

In contrast, artificial substrates such as collagen, generic Hemotek membranes, Parafilm^®^, or porcine gut yield very low or negligible feeding success in *Lu. Longipalpis*.^20^ Other sand fly species, including *Lu. migonei*, *P. perniciosus*, *P. duboscqi*, and *P. argentipes*, display greater plasticity, with moderate to high engorgement rates on a wider range of membranes, including porcine gut and duck foot webbing.^20^ Additionally, experiments using frog skin as a feeding membrane were unsuccessful. To enhance membrane attractiveness and improve feeding success, Hošková et al.^20^ coated the outer surface of both chicken skin and synthetic membranes with coagulated blood plasma (CBP) before offering them to *S. minuta* and other species exhibiting low feeding rates. Although this treatment significantly improved feeding success in most species tested, no increase in blood-feeding rates was observed *in Lu. longipalpis* fed through a synthetic membrane.

Unlike sand flies, culicids such as *Aedes aegypti* and *Culex quinquefasciatus* feed efficiently on synthetic membranes, particularly Parafilm^®^, which is considered the gold standard for these species and yields satisfactory performance in terms of engorgement, fecundity, and egg hatch, while collagen and latex result in inferior outcomes.^28^ Similarly, ixodid ticks such as *Amblyomma spp.* can engorge on membrane-based artificial feeding systems (AFS), although still at levels considerably lower than those achieved on live hosts.^29^

The present study confirmed this trend: females fed on hamsters displayed the highest engorgement rates (88.8%), reinforcing the preference of *Lu. longipalpis* for live hosts. This observation is consistent with previous investigations, which have shown that *in vivo* feeding consistently outperforms *in vitro* feeding due to the combined influence of host-derived chemical, thermal, and tactile cues that are difficult to replicate artificially.^16^ ^,^ ^22^ Among the membranes tested, both CHK (72.34%) and hydrated collagen treated with plasma gel (72.08%) supported efficient feeding, whereas untreated hydrated collagen membranes performed significantly worse (54.8%). CHK traditionally considered the “gold standard” for *Lu. longipalpis*, maintained consistently high feeding rates. However, the use of CHK presents important limitations, including the need for a larger number of animals for experimentation, an increased risk of contamination associated with its skin microbiota, and the requirement for storage at -20ºC for a limited period. In addition, its cost-benefit ratio is lower than that of collagen membranes, which can be stored at room temperature for an indefinite period. This finding suggests that although none of the artificial membranes achieved the ingestion levels observed in IVH, they performed equivalently among themselves, reinforcing their potential as alternatives to the use of vertebrate hosts in laboratory experiments, in line with more ethical and sustainable practices in entomological research.

The addition of coagulated plasma to hydrated collagen membranes markedly improved feeding success, raising performance to levels comparable with avian membranes. This effect, previously described as species- and membrane-dependent, appears to be beneficial for sand flies.^20^ In contrast, untreated collagen membranes yielded lower feeding success, in line with previous reports of synthetic membrane rejection by *Lu. Longipalpis*.^20^ Taken together, these findings reinforce the advantage of animal-derived membranes over purely synthetic ones for colony maintenance of this species. Taken together, these results indicate that both CHK and COL+PG treatments yielded similar feeding performance and represent viable alternatives to live hosts for blood feeding in *Lu. longipalpis*. Moreover, COL+PG, being an artificial membrane, can replace chicken skin as a feeding membrane for *Lu. longipalpis* in artificial feeding systems.

In recent years, image analysis techniques have emerged as innovative, non-invasive, and highly accurate alternatives for quantifying blood ingestion. Digital image processing has shown great promise in estimating blood meal volume, overcoming limitations of traditional approaches and enabling more efficient monitoring of vector feeding behaviour.^30^ In sand fly physiology and parasite-vector interaction studies, blood meal quantification is frequently required. Although haemoglobin dosage remains the most widely used method, it presents several operational limitations, including difficulties in processing large sample sizes and reduced reproducibility. Moreover, haemoglobin quantification relies on toxic reagents such as potassium cyanide,^31^ ^,^ ^32^ ^,^ ^33^ which pose significant biological and environmental risks.

In the present study, 7.2% of females fed through CHK membranes exhibited abdominal distension with a pale appearance, suggesting the ingestion of fluid or secretions containing little or no erythrocytes (Fig. 4). This phenotype has been consistently observed in females fed through CHK membranes in our laboratory. Haemoglobin quantification revealed that these individuals had ingested only small amounts of blood and were often classified as either poorly fed or unfed (data not shown). The implementation of image-based analysis in the present study minimised this source of bias, enabling more accurate detection of blood meals. Moreover, the permanent digital record of the specimens allows the raw data to be re-examined whenever necessary, thereby enhancing analytical transparency and reproducibility. Additionally, improvements in image acquisition (achieved through the use of a higher-resolution camera) allowed refinement of the image-processing workflow previously described by Costa et al.^24^ These advances also supported the development of a semi-automated ImageJ macro, which streamlined the analytical procedure and optimised the estimation of blood meal size across large numbers of insects.

*In vitro* feeding using hydrated collagen membranes and treated with plasma gel not only achieved feeding rates comparable with those obtained with CHK (~72%) but was also fully compatible with image analysis-based estimation of ingested volume. This combination represents a promising alternative to the use of vertebrate hosts for maintaining laboratory colonies of *Lu. longipalpis*. Colony maintenance is essential for both physiological experiments and parasite-vector interaction studies and depends directly on the availability of suitable blood sources. Multiple factors influence blood feeding in medically important dipterans, including genetic traits, host odours, and the release of aggregation pheromones by females.^34^ Aggregation effects have been described in *P. papatasi* ^35^ and *Lu. longipalpis*, as well as in other haematophagous arthropods such as ceratopogonids, simuliids, and *Amblyomma* ticks.^36^ ^,^ ^37^ ^,^ ^38^ ^,^ ^39^ Denlinger et al.^15^ demonstrated that larger feeding groups achieve higher engorgement rates both *in vivo* and *in vitro*, a phenomenon attributed to aggregation behaviour. The presence of males further enhances female feeding success through the release of pheromones that increase attraction to the blood source.^40^ ^,^ ^41^ In addition, carbon dioxide is widely recognised as a potent attractant for sand flies and other haematophagous dipterans.^42^ ^,^ ^43^ Together, these behavioural factors provide important insights into the mechanisms underlying blood-feeding success and help explain why plasma-treated membranes and host-associated chemical cues markedly improve feeding efficiency in artificial systems. By highlighting the synergistic effects of chemical, and environmental stimuli, these findings reinforce the potential of refining artificial feeding methods to reduce reliance on vertebrate hosts and to advance experimental studies of sand fly biology and vector-parasite interactions.

The implementation of artificial feeding as an alternative to the use of live hosts for the maintenance of laboratory sand fly colonies must take into account that the colonisation of these vectors remains restricted to a relatively small number of specialised laboratories.^23^ Although *Lu. longipalpis* is one of the main experimental models used in leishmaniasis research, information regarding physiological changes and the cumulative effects of infection over successive gonotrophic cycles remains limited, largely due to the high mortality observed in females after the first oviposition.^44^ This scenario contrasts with that of mosquitoes, whose laboratory rearing has been refined for more than a century. Currently, numerous mosquito species can be maintained for many generations using artificial feeding systems and a variety of blood sources, including isolated blood components such as plasma supplemented with phagostimulants.^45^ Consequently, the effects of different dietary compositions on feeding behaviour, reproduction, and overall biological performance have been extensively documented in mosquitoes.^46^ ^,^ ^47^ ^,^ ^48^ ^,^ ^49^ ^,^ ^50^

Accordingly, the integration of image analysis methodologies with plasma-treated collagen membranes represents a viable, safe, and ethically preferable strategy for experimental assay with *Lu. longipalpis*. Beyond reducing dependence on animal-derived membranes, this approach addresses longstanding methodological limitations in artificial blood feeding and blood meal quantification and enhances experimental reproducibility.

## SUPPLEMENTARY MATERIALS

Supplementary material

## Data Availability

After publication the data will be available on demand to corresponding author.

## References

[1] Galati EAB, Andrade AJ, Perveen F, Loyer M, Vongphayloth K, Randrianambinintsoa FJ (2025). Phlebotomine sand flies (Diptera, Psychodidae) of the world. Parasit Vectors.

[2] Maroli M, Feliciangeli MD, Bichaud L, Charrel RN, Gradoni L (2013). Phlebotomine sandflies and the spreading of leishmaniases and other diseases of public health concern. Med Vet Entomol.

[3] Modi GB, Tesh RB (1983). A simple technique for mass rearing *Lutzomyia longipalpis* and *Phlebotomus papatasi* (Diptera: Psychodidae) in the laboratory. J Med Entomol.

[4] Lainson R, Ward RD, Shaw JJ (1977). Experimental transmission of *Leishmania chagasi* by the sandfly *Lutzomyia longipalpis*. Nature.

[5] Killick-Kendrick R (1999). The biology and control of phlebotomine sand flies. Clin Dermatol.

[6] Boité MC, Mauricio IL, Miles MA, Cupolillo E (2012). New insights on taxonomy, phylogeny and population genetics of *Leishmania* (Viannia) parasites based on multilocus sequence analysis. PLoS Negl Trop Dis.

[7] Ready PD (2013). Biology of phlebotomine sand flies as vectors of disease agents. Annu Rev Entomol.

[8] Andrade-Filho JD, Scholte RGC, Amaral ALG, Shimabukuro PHF, Carvalho OS, Caldeira RL (2017). Occurrence and probability maps of *Lutzomyia longipalpis* and *Lutzomyia cruzi* in Brazil. J Med Entomol.

[9] Borges DA, Molina SMG, Pinto MC, Galati EAB, Cesário M, Ortiz DGS (2017). First record of *Lutzomyia longipalpis* on the trinational frontier of south-western Amazonia. J Med Entomol.

[10] Cavalcante KS, Galardo AKR, Saraiva JF, Barbosa TA, Galeno EO, Nunes MCDL (2024). The first identification of *Lutzomyia longipalpis* (Lutz & Neiva, 1912) in Macapá, Amapá. Rev Saude Publica.

[11] Lainson R, Rangel EF (2005). *Lutzomyia longipalpis* and the eco-epidemiology of American visceral leishmaniasis, with particular reference to Brazil - A review. Mem Inst Oswaldo Cruz.

[12] Galati EAB, Rangel EF, Shaw JJ (2018). Brazilian sand flies.

[13] Lima-Junior MSDC, Almeida PS, Silva JO, Silva RA, Faccenda O, Coelho DVBSA (2021). Sand fly fauna, spatial distribution of *Lutzomyia longipalpis*, and climate factors in Dourados, Brazil. J Med Entomol.

[14] Missawa NA, Lorosa ES, Dias ES (2008). Feeding preference of *Lutzomyia longipalpis* (Lutz & Neiva, 1912) in transmission area of visceral leishmaniasis in Mato Grosso. Rev Soc Bras Med Trop.

[15] Denlinger DS, Li AY, Durham SL, Lawyer PG, Anderson JL, Bernhardt SA (2016). Comparison of *in vivo* and *in vitro* methods for blood feeding of *Phlebotomus papatasi* in the laboratory. J Med Entomol.

[16] Volf P, Volfova V (2011). Establishment and maintenance of sand fly colonies. J Vector Ecol.

[17] Sánchez Uzcátegui YDV, Santos EJM, Matos ER, Silveira FT, Santos TV, Póvoa MM (2022). Artificial blood-feeding of phlebotomines: is it time to repurpose biological membranes in light of ethical concerns?. Parasit Vectors.

[18] Ready PD (1978). The feeding habits of laboratory-bred *Lutzomyia longipalpis*. J Med Entomol.

[19] Ward RD, Lainson R, Shaw JJ (1978). Some methods for membrane feeding of laboratory-reared neotropical sandflies. Ann Trop Med Parasitol.

[20] Hošková A, Vojtková B, Stejskalová M, Polanská N, Jančářová M, Costa LM (2025). Evaluation of various membranes for blood-feeding in nine sand fly species. Parasit Vectors.

[21] Sádlová J, Homola M, Myskova J, Jančářová M, Volf P (2018). Refractoriness of *Sergentomyia schwetzi* to *Leishmania* spp. is mediated by the peritrophic matrix. PLoS Negl Trop Dis.

[22] Rowton ED, Dorsey KM, Armstrong KL (2008). Comparison of *in vitro* versus *in vivo* blood-feeding methods for maintenance of colonized *Phlebotomus papatasi*. J Med Entomol.

[23] Lawyer P, Killick-Kendrick M, Rowland T, Rowton E, Volf P (2017). Laboratory colonization and mass rearing of phlebotomine sand flies. Parasite.

[24] Costa LM, Sant’Anna MRV, Generoso LA, da Cruz GP, Pessoa GCD, Araujo RN (2026). A non-destructive image-based approach to quantify blood meal size in *Lutzomyia longipalpis*. Mem Inst Oswaldo Cruz.

[25] Alexander B, Carvalho RL, McCallum H, Pereira MH (2002). Role of the domestic chicken in the epidemiology of urban visceral leishmaniasis in Brazil. Emerg Infect Dis.

[26] Casanova C, Andrighetti MTM, Sampaio SMP, Marcoris MLG, Colla-Jacques FE, Prado AP (2013). Larval breeding sites of *Lutzomyia longipalpis* in urban endemic areas. PLoS Negl Trop Dis.

[27] Sant’Anna MRV, Nascimento A, Alexander B, Dilger E, Cavalcante RR, Diaz-Albiter HM (2010). Chicken blood provides a suitable meal for *Lutzomyia longipalpis*. Parasit Vectors.

[28] Dias LS, Caldeira JC, Bauzer LGSR, Lima JBP (2020). Assessment of synthetic membranes for artificial blood feeding of Culicidae. Insects.

[29] Sebastian PS, Król N, Novoa MB, Nijhof AM, Pfeffer M, Nava S (2023). Preliminary Study on artificial versus animal-based feeding systems for *Amblyomma* ticks. Microorganisms.

[30] Janson KD, Carter BH, Jameson SB, Verges JE, Dalliance ES, Royse MK (2023). Development of an automated biomaterial platform to study mosquito feeding behavior. Front Bioeng Biotechnol.

[31] Ranzani-Paiva MJT, Pádua SB, Tavares-Dias M, Egami MI (2013). Métodos para análise hematológica em peixes.

[32] Shah V, Shah B, Puranik G (2011). Evaluation of non-cyanide methods for hemoglobin estimation. Indian J Pathol Microbiol.

[33] Malin MJ, Fan SS, Benezra J (1992). Mechanism of automated alkaline methods for determination of hemoglobin. Anal Chim Acta.

[34] Francisco CM, Silva WF (2019). Fatores que influenciam no repasto sanguíneo de mosquitos. Visa Debate.

[35] Schlein Y, Yuval B, Warburg A (1984). Aggregation pheromone released from palps of feeding females *Phlebotomus papatasi* (Psychodidae). J Insect Physiol.

[36] Norval RAI, Andrew HR, Yunker CE (1989). Pheromone mediation of host selection in ticks. Science.

[37] Kelly DW, Dye C (1997). Pheromones and aggregation dynamics of *Lutzomyia longipalpis*. Anim Behav.

[38] McCall PJ, Lemoh PA (1997). Evidence for the invitation effect during bloodfeeding by blackflies. J Insect Behav.

[39] Tripet F, Aboagye-Antwi F, Hurd H (2008). Ecological immunology of mosquito-malaria interactions. Trends Parasitol.

[40] Ward RD, Hart DT (1989). Leishmaniasis.

[41] Morton IE, Ward RD (1989). Laboratory response of female *Lutzomyia longipalpis* to host and pheromone source. Med Vet Entomol.

[42] Gibson G, Torr SJ (1999). Visual and olfactory responses of haematophagous Diptera. Med Vet Entomol.

[43] Pinto MC, Campbell-Lendrum DH, Lozovei AL, Teodoro U, Davies CR (2001). Phlebotomine sandfly responses to carbon dioxide and human odour. Med Vet Entomol.

[44] Moraes CS, Aguiar-Martins K, Costa SG, Bates PA, Dillon RJ, Genta FA (2018). Second blood meal by female *Lutzomyia longipalpis*: enhancement by oviposition and its effects on digestion, longevity, and *Leishmania* infection. Biomed Res Int.

[45] Hari I, Gaikwad P, Ray SK, Jenish JA, Priya PT, Alagarasu K (2026). Establishing and sustaining mosquito colonies: insights into morphology, bionomics, and advances in the rearing of *Anopheles*, *Culex*, and *Aedes*. Front Insect Sci.

[46] Woke PA (1937). Comparative effects of blood of vertebrates on egg production of *Aedes aegypti*. Am J Trop Med Hyg.

[47] Greenberg J (1951). Some nutritional requirements of adult mosquitoes for oviposition. J Nutr.

[48] Spielman A, Wong J (1974). Dietary factors stimulating oogenesis in *Aedes aegypti*. Biol Bull.

[49] Harrington LC, Edman JD, Scott TW (2001). Why do female *Aedes aegypti* feed preferentially on human blood?. J Med Entomol.

[50] Harrison RE, Brown MR, Strand MR (2021). Blood components affect egg formation in mosquitoes. Parasit Vectors.

