# Supplementary material for: Application of image analysis in the evaluation of blood feeding by Lutzomyia longipalpis using an artificial feeding system with synthetic membranes
Source: Mem Inst Oswaldo Cruz. 2026 Oct 16;121:e260065. doi: 10.1590/0074-02760260065 (PMC13673989; doi:10.1590/0074-02760260065)
Supplement: Supplementary file 1 [file 1678-8060-mioc-121-e260065-s1.pdf]

(I)

Script (macro) developed in ImageJ to import images, define the region of interest, apply thresholding to isolate the red area, and automatically measure its area, with the option for manual adjustments during processing:

"// Initialise path0 to avoid errors when comparing with empty string var path0 = ""; // Check if path0 is empty or undefined if (path0 == "") {// If path0 was not defined, open the dialog
path0 = File.openDialog("Choose an image file");}

// Print the path print(path0);

// Replace backslashes with normal slashes path = replace(path0, "\\", "/");

// Find the position of the last slash lastSlashIndex = lastIndexOf(path, "/");

// Extract the filename (substring after the last slash) fileName = substring(path, lastSlashIndex + 1);

// Find the position of the last dot lastSlashIndex0 = lastIndexOf(path, ".");

// Extract the file extension
ext_arq = substring(path, lastSlashIndex0 + 1); print(ext_arq);

// Remove the extension from the filename arq = fileName.replace("." + ext_arq, "");

// Print the filename
print(fileName); // "1-5 Hamster.CR2"

run("Bio-Formats Importer", "open=[" + path + "] " + "autoscale color_mode=Default view=Hyperstack stack_order=XYCZT");

// Draw rectangles makeRectangle(1350, 600, 2724, 1998);

// Create dialog with title "Confirmation" Dialog.create("Confirmation");

// Function that displays the confirmation window function showDialog() {
// Create dialog with title "Confirmation" Dialog.create("Confirmation");

// Add message to the dialog with line break
Dialog.addMessage("Check if the selected area is correct. \n \nIf not, check the 'Change' option!");

// Add checkbox for "Change" option Dialog.addCheckbox("Change", false);

// Add a numeric field for editing time (in seconds) Dialog.addNumber("Editing time (seconds):", 7); // Default is 10 seconds

// Show the dialog Dialog.show();

// Get dialog values
alterarEscolhido = Dialog.getCheckbox();
tempoEdicao = Dialog.getNumber(); // Editing time in seconds
// Convert checkbox response to boolean if (alterarEscolhido == true) {escolhaBooleano = true; // User chose "Change"} else {escolhaBooleano = false; // User did not choose "Change"}

// Print values
print("Boolean choice (Change):", escolhaBooleano); print("Editing time (seconds):", tempoEdicao);

// If the user chose to change, wait and show dialog again if (alterarEscolhido == true) {print("Waiting for " + tempoEdicao + " seconds for editing..."); wait(tempoEdicao * 1000); print("Editing time is over. Showing confirmation window again..."); showDialog(); // Call again }}

// Initialise first dialog showDialog();

// Duplicate layer run("Duplicate...", "duplicate");
// Convert to RGB run("RGB Colour");

```
// Replace red with magenta run("Replace Red with Magenta");
// Convert to CMYK run("RGB to CMYK");
run("Duplicate...", "duplicate channels=2"); run("8-bit");
//run("Threshold...");
run("Threshold...");

// Create dialog with title "Confirmation" Dialog.create("Confirmation");

function showDialog01() {// Create dialog with title "Confirmation" Dialog.create("Confirmation");

// Add message
Dialog.addMessage("Make your changes in Threshold.\n \nWhen finished, wait for this window to appear again and click 'Done'.");

// Checkbox "Done" Dialog.addCheckbox("Done", false);

// Editing time
Dialog.addNumber("Editing time (seconds):", 7);

// Show dialog Dialog.show();

// Get values
var prontoEscolhido = Dialog.getCheckbox(); var tempoEdicao = Dialog.getNumber();

var prontoBooleano = prontoEscolhido;

print("Boolean choice (Done):", prontoBooleano);
print("Editing time (seconds):", tempoEdicao);

if (prontoEscolhido == false) {
print("Waiting for " + tempoEdicao + " seconds for editing..."); wait(tempoEdicao * 1000);

print("Editing time is over. Showing confirmation window again..."); showDialog01();
}
}

// Initialise first dialog showDialog01();

run("Make Binary"); doWand(0, 0);
// Initialise magic wand tool setTool("wand");

// Create dialog with title "Confirmation" Dialog.create("Confirmation");

function showDialog0() { Dialog.create("Confirmation");

Dialog.addMessage("Holding Shift, click the black areas.\n \nWhen finished, wait for this window to reappear and click 'Done'.");

Dialog.addCheckbox("Done", false);

Dialog.addNumber("Editing time (seconds):", 7);
Dialog.show();

var prontoEscolhido = Dialog.getCheckbox(); var tempoEdicao = Dialog.getNumber();

var prontoBooleano = prontoEscolhido;

print("Boolean choice (Done):", prontoBooleano); print("Editing time (seconds):", tempoEdicao);

if (prontoEscolhido == false) {
print("Waiting for " + tempoEdicao + " seconds for editing..."); wait(tempoEdicao * 1000);
```

```
print("Editing time is over. Showing confirmation window again..."); showDialog0();
}
}

// Initialise first dialog showDialog0();

// Manage and measure ROIs roiManager("Add"); roiManager("Select", 0);
run("Set Measurements...", "area redirect=None decimal=4"); roiManager("Measure");"
```

(II)

README file describing the workflow, requirements, and usage instructions for the semi-automated macro developed in ImageJ to estimate the blood-fed area in sandfly images through colour conversion, threshold segmentation, and user-assisted region selection.

README — CALCULATE TO RED AREA.ijm

1. Purpose

This ImageJ/Fiji macro provides a semi-automated workflow to estimate the area of ingested blood (red colour ROI) by sandflies in a digital photograph. The macro combines colour-space conversion, threshold-based segmentation, and user-assisted region selection to produce reproducible area measurements.

2. Tested Environment (Reproducibility)

Tested on:

Fiji (ImageJ 1.54p)

Java 1.8.0_322 (64-bit)

Operating System: Windows 64-bit Test Date 23/02/2026

3. Required Dependencies

a. Fiji (https:/imagej.net/software/fiji/downloads)

b. RGB to CMYK plugin (https://imagej.net/ij/plugins/cmyk/index.html)

c. Bio-Formats Importer (included in Fiji by default)

4. Macro Workflow

a. Opens a single image selected by the user (via Bio-Formats Importer).

b. Defines an initial rectangular ROI (Region of Interest), adjustable by the user.

c. Duplicates the ROI and performs colour processing:

- Converts to RGB

- Replaces Red with Magenta

- Converts RGB → CMYK

- Duplicates CMYK Channel 2

- Converts to 8-bit grayscale

d. Opens the Threshold tool for interactive adjustment.

e. Converts the thresholder image to a binary mask.

f. Activates the Wand tool for manual region selection (hold SHIFT to add regions).

g. Stores the final ROI in ROI Manager.

h. Measures area (Analyse > Measure).

i. Writes results to the Results table.

5. Interactive Steps

ROI Confirmation:

A dialog allows the user to reposition or resize the rectangular ROI before proceeding. Threshold Adjustment: The Threshold window opens. The user fine-tunes segmentation and confirms when satisfied. Wand Selection: The Wand tool is activated. SHIFT-click to select multiple regions. Confirm when finished.

6. Output

a. Final abdomen ROI stored in ROI Manager.

b. Area measurement written to the Results table.

c. Units depend on calibration settings.

7. Image Requirements

Required:

• Single RGB image readable by Fiji (TIFF, JPEG, PNG, RAW via Bio-Formats).

• Adequate contrast between abdomen and background.

Strongly Recommended:

• Stable illumination.

• Uniform background.

• Minimal shadows and reflections.

• Consistent magnification and specimen orientation.

8. ROI Coordinates (Important)

The macro starts with the following rectangle coordinates:

makeRectangle(1350, 600, 2724, 1998);

If your images differ in size, adjust these coordinates accordingly.

9. Calibration

If spatial calibration is set (Analyse > Set Scale), the output area will be reported in calibrated units (e.g., mm^2 or μm^2). If not calibrated, the area will be reported in $pixel^2$.

10. Important Considerations

• Segmentation depends on colour contrast.

• Requires user intervention.

• Not fully automated.

• ROI coordinates may need adjustment for different datasets.
