# Supplementary material for: Application of image analysis in the evaluation of blood feeding by Lutzomyia longipalpis using an artificial feeding system with synthetic membranes
Source: Mem Inst Oswaldo Cruz. 2026 Oct 16;121:e260065. doi: 10.1590/0074-02760260065 (PMC13673989; doi:10.1590/0074-02760260065)
Supplement: Peer review history

# Reviewer report: FIRST REVIEW ROUND - REVIEWERS’ COMMENTS

## REVIEWER #1

This study introduces a new method for determining the blood meal size in sand flies using image analysis and compares the feeding behaviour of L. longipalpis females in vivo versus in vitro, showing that a hydrated collagen membrane treated with blood plasma achieves the same feeding rate as chicken skin, both approaching the feeding rate on hamsters (72 vs 89%).

Formally, the article is well-written, with all the methodological details and high-quality images, and I appreciate the inclusion of the ImageJ macro as supplementary material.

I recommend only minor revisions and a more precise interpretation of the data in the discussion.

• Page 3, line 9: Instead of the first reference focusing on a limited geographical area, I would recommend citing the recent review by Galati et al. (2025), DOI:10.1186/s13071-025-06748-5.

• Page 5, line 8: italicise Mesocricetus auratus

• Page 5, lines 43-47, The sentence “For both colony maintenance and experimental feeding, hamsters were previously anaesthetised“ is redundant (this information is provided above).

• Page 6, line 34: Check spelling of the sentence.

• Link to Figure 3 is missing.

• Page 9, lines 32–39: This sentence should be moved to the Discussion.

• Page 10, lines 17-22: Again, the sentence should be moved to the Discussion.

• Page 11, lines 15-16:“ whereas dry untreated collagen membranes performed significantly worse“ - Shouldn’t that be “untreated hydrated collagen membranes”?

• Page 11-12: To keep the discussion on quantification of blood meal balanced, it should be noted that colorimetric determination of haemoglobin content has (despite the limitations described) a significant advantage over the weighing method or the image analysis method used here: it can clearly distinguish haemoglobin content from water content which is important for certain experiments (plasma fed females cannot simply be considered a false negative result).

• Page 12: The sentence “False-negative classifications may also occur when females ingest plasma or plasma-diluted blood but are erroneously recorded as unfed.“ is repeated twice here.

• Page 12 line 30: IIn vitro

• Page 13 lines 13-20: It should be noted that this phenomenon has so far been described in mosquitoes, but not in sandflies.

• Page 13, lines 21-24: „Accordingly, the integration of image analysis methodologies with plasma treated collagen membranes represents a viable, safe, and ethically preferable strategy for maintaining laboratory colonies of Lu. longipalpis.“ I do not suppose that image analysis can be useful in routine colony maintenance. A clearer distinction needs to be made here between use for experimental purposes and for breeding.

• In the discussion on the possibility of using artificial feeding for the long-term rearing of sand flies, it is necessary to cite studies demonstrating that there is no long-term effect of artificial feeding on fecundity and mortality of females. If no such study exists, this should be emphasized throughout the abstract and discussion.

## REVIEWER #2

The study evaluated the feeding performance of Lu. longipalpis using different food sources [hamster (control), chick membrane, skin, hydrated collagen membrane treated with plasma gel, and untreated hydrated collagen membrane]. Blood meal size was quantified through image analysis, following the methodology previously described by the authors (Costa et al., 2026). The manuscript is well written, with a clear and concise methodology.

a) abstract: line 4. Change to “The primary (or principal) vector of Leishmania infantum, the etiological agent of visceral leishmaniasis in the Americas.

line 36-37. The phrase “while image analysis provides a non-invasive, reproducible, and scalable method for blood quantification” appears to be part of the conclusion of the previous study (Costa et al., 2026), which validated a non-destructive image-based method for estimating blood intake in Lutzomyia longipalpis. Is this correct? If so, it should be revised.

b) Introduction: Page 3

line 7. Update the information. According Galati et al. 2025, they are 1063 sand fly species, with 549 for New World. Galati et al. Parasites & Vectors (2025) 18:220

https://doi.org/10.1186/s13071-025-06748-5.

line 10. The authors could expand on the sentence, as few people are familiar with the term “Harara”. “can cause allergic reactions such as Hararaa, a severe, chronic papular urticaria (hives) reaction to the bites of sand flies”.

line 12. “The primary (or principal) vector of Leishmania infantum, the etiological agent of visceral leishmaniasis in the Americas.”

line 43-46. Please provide a reference.”More recently, duck foot interdigital membranes have shown comparable performance, representing a viable alternative that reduces the need for neonatal chicks”.

line 48. Include a reference Sánches Uzcátegui et al. 2022. This article tests pig intestines for the first time in this type of study.

c) Methodology, results and discussion: The methodology is well described and reproducible. Did the authors verify feeding time using the different methodologies employed? This would be interesting data to determine if there are any difficulties/ease for the insect to feed in vivo or on membranes.

Page 12, line 31 There is an error in “IIn vitro”.

Nothing to add regarding the references, figures, and graphs.

## REVIEWER #3

Reviewer Report (for Memórias do Instituto Oswaldo Cruz – MIOC)

Manuscript ID: MIOC-2026-0065

Title: Application of image analysis in the evaluation of blood feeding by Lutzomyia longipalpis using an artificial feeding system with synthetic membranes

This manuscript addresses an important methodological bottleneck in sand fly research: improving artificial feeding and implementing image-based, non-destructive blood-meal quantification. The comparison of membranes (chick skin vs hydrated collagen ± plasma gel) and the Fiji/ImageJ workflow are timely and potentially valuable for colony maintenance and experimental studies.

Introduction:

The Introduction would benefit from being more focused and concise. At present, it feels somewhat long and includes some repeated background points (e.g., multiple statements about ethical/logistical limitations of in vivo feeding and the general importance of membrane choice and artificial feeding).

Methods:

Page 4 line 51: Explain the expression “closed colony” as follow: A closed breeding colony of Lu. longipalpis (originating from Teresina, Piauí, Brazil) has been maintained in the insectary of the Laboratory of Haematophagous Insect Physiology (ICB–UFMG) since 2009 under controlled conditions (25 ± 1 °C; 80–95% relative humidity).

Page 5 Line 8: Format species name in italic.

Feeding assays: The groups acronyms are not self-explanatory and can make interpreting the results difficult. Suggest the authors to change the acronyms and include a sentence in the methodology: Groups were: IVH (in vivo hamster), CHK (chick skin membrane), COL+PG (hydrated collagen treated with plasma gel), COL (hydrated collagen untreated), and UNF (unfed control).

Explain that the negative control “unfed” group was used to establish a criterion of abdominal area to classify the insects as engorged or unfed. The criterion uses the negative control mean + 2 SD threshold. Ensure it is described clearly as an analytic reference and that sample sizes used for thresholding.

Page 5 Line 35: Abbreviate the genus Mesocricetus, as it is already written in full on line 8.

Page 5 Line 40: Change One to A group

Page 5 Line 40: Include how many sand flies were used and was only females or males together. The number of insects used should be described in each group in the methodology.

Page 5 Line 54: The sentence of 12h starved is already explained in Feeding assay

Page 5 Line 57: Is there any ethics license for the human blood used?

It would improve clarity if the Methods description of all feeding conditions followed the same internal structure within the “Feeding assays” section. The authors introduce the subsections “Live host” and “Artificial feeder,” but the subsequent description of membrane treatments (chick skin vs. collagen ± plasma gel) is presented as a separate block rather than as a continuation of the artificial-feeder workflow. As a result, readers must piece together which procedures correspond to each experimental group and how many sand flies were used per feeding condition. I recommend reorganizing this part of the Methods so that each feeding group is described in a parallel format (e.g., group definition → number of females/males per replicate → feeding duration/temperature → membrane preparation → post-feeding handling), or alternatively nesting “Membrane treatments” as a sub-subsection under “Artificial feeder.” This would make it much easier for readers to understand the experimental groups and the sample size used in each feeding procedure.

Page 6 Line 49: Avoid abbreviations that may overlap with widely used terms. “ABS” is commonly read as “absorbance,” whereas “RCS” is not intuitive or self-explanatory to readers. Use the suggestion below:

ABS (whole abdominal area) → A_abd (abdominal area) or AbdA (Abdominal Area)

RCS (red-coloured blood area) → A_blood (blood area) or RCS → BldA (Blood Area)

Include the following sentence in the section Image acquisition: Blood meal size was estimated by image analysis using two ROIs: abdominal area (A_abd) and blood/red area (A_blood).

Page 6 line 32 - The text states that dry vs hydrated collagen differed significantly but reports “t-test p>0.005” Please correct the p-value direction/threshold p<0.05.

Page 7 – Cite figure 3 in the methods.

Results:

Page 8, lines 41. “17 of 235 females (0.03%) were reclassified as engorged.” This percentage is mathematically incorrect (17/235 ≈ 7.2%). The authors must correct.

Page 9, lines 47 In the Results, the correlation between ABS and RCS (R² = 0.85) is described and then referenced to “Fig. 5,” but Fig. 5 is the barplot of feeding percentages; the regression is shown in Fig. 7. Please correct the figure reference(s) to avoid reader confusion.

Discussion

There are sections of justification and introduction in the Discussion that could be removed.

Some paragraphs lack coherence and connection with each other.

On page 12, there is a double space between paragraphs.

The sentence between lines 14 and 21 on page 13 would be better placed after the end of the first paragraph on page 12, as it would also help explain the importance of including females that fed only on plasma and few erythrocytes.

It is also necessary to include in the Discussion the rationale for adding plasma gel to the membrane.

Page 12 line 31“IIn vitro feeding…” (extra “I”).

Decimal punctuation: Please standardize decimal separators in English text/tables/figures. The manuscript mixes “0.84” and “0,84” and “p<0,05.” Use “0.84” and “p<0.05” consistently.

Figure legends should have a Title (a short, informative title that captures the whole figure), Groups/conditions, description of each panel (A, B, C…), briefly methodology, definition of all experimental groups/conditions and expand any abbreviations used in the figure, symbols/annotations, sample size (n), replicates and statistics.

Figure 1 could be moved to Supplementary materials

Figures 1–4 are illustrative; consider adding “representative images”

Figure 7 legend - should include key regression statistics (n, R², p-value, and ideally regression equation and CI).

References: include DOI

## AUTHORS’ RESPONSE TO THE REVIEWERS

Dear Editor,

We have carefully considered the reviewer’s comments and revised the manuscript in accordance with their suggestions.

Yours sincerely,

## REVIEWER COMMENTS

### Reviewer 1:

This study introduces a new method for determining the blood meal size in sand flies using image analysis and compares the feeding behaviour of L. longipalpis females in vivo versus in vitro, showing that a hydrated collagen membrane treated with blood plasma achieves the same feeding rate as chicken skin, both approaching the feeding rate on hamsters (72 vs 89%). Formally, the article is well-written, with all the methodological details and high-quality images, and I appreciate the inclusion of the ImageJ macro as supplementary material. I recommend only minor revisions and a more precise interpretation of the data in the discussion.

Response: We thank the reviewer for their valuable comments and suggestions.

• Page 3, line 9: Instead of the first reference focusing on a limited geographical area, I would recommend citing the recent review by Galati et al. (2025), DOI:10.1186/s13071-025-06748-5.

a) We agree with the reviewer that the review by Galati et al. (2025) provides a more current overview of the subject. Therefore, we have replaced the citation of Shimabukuro and Galati (2011) with Galati et al. (2025) in the reference list. In addition, the citation of Young and Duncan (1994) has been replaced with Maroli et al. (2013). Based on these updated references, we have revised the first sentence of the Introduction as follows:

“Currently, 1063 sand fly species are described, of which 82 species belong to Lutzomyia, including more than 40 proven Leishmania vectors (1,2)”

• Page 5, line 8: italicise Mesocricetus auratus.

b) We have italicized Mesocricetus auratus throughout the manuscript.

• Page 5, lines 43-47, The sentence “For both colony maintenance and experimental feeding, hamsters were previously anaesthetised“is redundant (this information is provided above).

c) The sentence below has been removed from the manuscript:

“For both colony maintenance and experimental feeding, hamsters were previously anaesthetised”

• Page 6, line 34: Check spelling of the sentence.

d) The sentence below has been rewritten as follows:

“Thus, in present study was used only hydrated collagen membrane”

• Link to Figure 3 is missing.

e) Indeed, figure 3 was not cited in the text. The figure has now been cited on page 8, between lines 3 and 7:

“The abdominal contour was then delineated after conversion to 8-bit grayscale, threshold adjustment, and automatic boundary detection using the Wand (tracing) tool (Fig. 3A-B)”

• Page 9, lines 32–39: This sentence should be moved to the Discussion.

f) We agree with the reviewer’s suggestion. This sentence has been moved to the Discussion section:

“This finding suggests that although none of the artificial membranes achieved the ingestion levels observed in IVH, they performed equivalently among themselves, reinforcing their potential as alternatives to the use of vertebrate hosts in laboratory experiments, in line with more ethical and sustainable practices in entomological research.”

• Page 10, lines 17-22: Again, the sentence should be moved to the Discussion.

g) We agree with the reviewer’s suggestion and have therefore moved this sentence to the Discussion section:

“Taken together, these results indicate that both CHK and COL+PG treatments yielded similar feeding performance and represent viable alternatives to live hosts for blood feeding in Lu. longipalpis. Moreover, COL+PG, being an artificial membrane, can replace chicken skin as a feeding membrane for Lu. longipalpis in artificial feeding systems”

• Page 11, lines 15-16: “whereas dry untreated collagen membranes performed significantly worse”- Shouldn’t that be “untreated hydrated collagen membranes”?

h) We agree with the reviewer. The correct term is “untreated hydrated collagen membranes”, and this has been corrected throughout the manuscript.:

“Among the membranes tested, both chick skin (72.34%) and hydrated collagen treated with plasma gel (72.08%) supported efficient feeding, whereas untreated hydrated collagen membranes performed significantly worse (54.8%).”

• Page 11-12: To keep the discussion on quantification of blood meal balanced, it should be noted that colorimetric determination of haemoglobin content has (despite the limitations described) a significant advantage over the weighing method or the image analysis method used here: it can clearly distinguish haemoglobin content from water content which is important for certain experiments (plasma fed females cannot simply be considered a false negative result).

i) We agree with the reviewer that this statement lacked sufficient context. Accordingly, this section of the Discussion has been rewritten as follows:

“In the present study, 7.2% of females fed through chick skin membranes exhibited abdominal distension with a pale appearance, suggesting the ingestion of fluid or secretions containing little or no erythrocytes (Fig. 4). This phenotype has been consistently observed in females fed through chick skin membranes in our laboratory. Hemoglobin quantification revealed that these individuals had ingested only small amounts of blood and were often classified as either poorly fed or unfed (data not shown).”

• Page 12: The sentence “False-negative classifications may also occur when females ingest plasma or plasma-diluted blood but are erroneously recorded as unfed.” is repeated twice here.

j) The sentence below has been removed from the manuscript.

False-negative classifications may also occur when females ingest plasma or plasma-diluted blood but are erroneously recorded as unfed.

• Page 12 line 30: IIn vitro

k) Ok, corrected.

• Page 13 lines 13-20: It should be noted that this phenomenon has so far been described in mosquitoes, but not in sandflies.

l) The reviewer is correct. To the best of our knowledge, we were unable to identify any published studies on sand flies evaluating the impact of a “plasma-based” diet on insect biology. The application of the methodologies proposed in the present manuscript is primarily intended for research purposes. We propose adding a brief paragraph to the Discussion section to contextualize our response by comparing the current state of knowledge regarding the biology and laboratory maintenance of sand flies and mosquitoes: “The implementation of artificial feeding as an alternative to the use of live hosts for the maintenance of laboratory sand fly colonies must take into account that the colonization of these vectors remains restricted to a relatively small number of specialized laboratories (23). Although Lu. longipalpis is one of the main experimental models used in leishmaniasis research, information regarding physiological changes and the cumulative effects of infection over successive gonotrophic cycles remains limited, largely due to the high mortality observed in females after the first oviposition (44). This scenario contrasts with that of mosquitoes, whose laboratory rearing has been refined for more than a century. Currently, numerous mosquito species can be maintained for many generations using artificial feeding systems and a variety of blood sources, including isolated blood components such as plasma supplemented with phagostimulants (45). Consequently, the effects of different dietary compositions on feeding behavior, reproduction, and overall biological performance have been extensively documented in mosquitoes (46-50).”

• Page 13, lines 21-24: “Accordingly, the integration of image analysis methodologies with plasma treated collagen membranes represents a viable, safe, and ethically preferable strategy for maintaining laboratory colonies of Lu. longipalpis.” I do not suppose that image analysis can be useful in routine colony maintenance. A clearer distinction needs to be made here between use for experimental purposes and for breeding.

m) We agree with the reviewer that this statement lacked clarity regarding the application of image analysis and the use of the treated membrane. Consequently, this section of the Discussion has been rewritten as follows:

“Accordingly, the integration of image analysis methodologies with plasma-treated collagen membranes represents a viable, safe, and ethically preferable strategy for experimental assay with Lu. longipalpis. Beyond reducing dependence on animal-derived membranes, this approach addresses longstanding methodological limitations in artificial blood feeding and blood meal quantification and enhances experimental reproducibility.”

• In the discussion on the possibility of using artificial feeding for the long-term rearing of sand flies, it is necessary to cite studies demonstrating that there is no long-term effect of artificial feeding on fecundity and mortality of females. If no such study exists, this should be emphasized throughout the abstract and discussion.

n) We were unable to identify any published studies reporting the maintenance of sand fly colonies under laboratory conditions using artificial feeding as the sole source of blood meals. Therefore, we reviewed excerpts from the abstract and discussion related to the application of artificial feeding for the maintenance of sand fly colonies.

Abstract:

“Collectively, this approach refines laboratory experiments with Lu. longipalpis and enhances methodological reliability in studies of sand fly biology and vector–parasite interactions”.

Discussão:

Please see answer “m”.

### Reviewer 2:

The study evaluated the feeding performance of Lu. longipalpis using different food sources [hamster (control), chick membrane, skin, hydrated collagen membrane treated with plasma gel, and untreated hydrated collagen membrane]. Blood meal size was quantified through image analysis, following the methodology previously described by the authors (Costa et al., 2026). The manuscript is well written, with a clear and concise methodology.

Response: We thank the reviewer for their valuable comments and suggestions.

• abstract: line 4. Change to “The primary (or principal) vector of Leishmania infantum, the etiological agent of visceral leishmaniasis in the Americas.

a) The text below has been corrected:

“However, artificial feeding systems for Lutzomyia longipalpis, the principal vector of Leishmania infantum, the etiological agent of visceral in the Americas, remain limited by low engorgement rates and methodological constraints in blood meal quantification”

• line 36-37. The phrase “while image analysis provides a non-invasive, reproducible, and scalable method for blood quantification” appears to be part of the conclusion of the previous study (Costa et al., 2026), which validated a non-destructive image-based method for estimating blood intake in Lutzomyia longipalpis. Is this correct? If so, it should be revised.

b) The text below has been rewritten, as follows:

“while image analysis provides a precise, reproducible, and practical method for blood meal quantification “.

• Introduction: Page 3 line 7. Update the information. According Galati et al. 2025, they are 1063 sand fly species, with 549 for New World. Galati et al. Parasites & Vectors (2025) 18:220. https://doi.org/10.1186/s13071-025-06748-5.

c) We agree with the reviewer that the review by Galati et al. (2025) provides a more current overview of the subject. Therefore, we have replaced the citation of Shimabukuro and Galati (2011) with Galati et al. (2025) in the reference list. In addition, the citation of Young and Duncan (1994) has been replaced with Maroli et al. (2013). Based on these updated references, we have revised the first sentence of the Introduction as follows:

“Currently, 1063 sand fly species are described, of which 82 species belong to Lutzomyia, including more than 40 proven Leishmania vectors (1,2)”

• line 10. The authors could expand on the sentence, as few people are familiar with the term “Harara”. “can cause allergic reactions such as Hararaa, a severe, chronic papular urticaria (hives) reaction to the bites of sand flies”.

d) As suggested by the reviewer, the information regarding Harara has been expanded in the manuscript:

“Some species also transmit bacteria, arboviruses, and can cause allergic reactions such as Harara, a severe chronic papular urticarial reaction induced by sand fly bites (3).”

• line 12. “The primary (or principal) vector of Leishmania infantum, the etiological agent of visceral leishmaniasis in the Americas.”

e) The text below has been corrected, as follows: 

“Lutzomyia longipalpis is the principal vector of Leishmania infantum, the etiological agent of visceral leishmaniasis in the Americas, transmitting Leishmania infantum between dogs and humans (4, 5)”

• line 43-46. Please provide a reference.”More recently, duck foot interdigital membranes have shown comparable performance, representing a viable alternative that reduces the need for neonatal chicks”.

f) Indeed, the statement in question was missing the corresponding reference [reference no. 20, Hošková et al. (2025)]. This citation has now been added to this sentence:

“have shown comparable performance, representing a viable alternative that reduces the need for neonatal chicks (20).”

• line 48. Include a reference Sánches Uzcátegui et al. 2022. This article tests pig intestines for the first time in this type of study.

g) The article is already included in the reference list of the manuscript. It corresponds to reference no. 17 and is cited in the final sentence of the second paragraph of the Introduction.

• Methodology, results and discussion: The methodology is well described and reproducible. Did the authors verify feeding time using the different methodologies employed? This would be interesting data to determine if there are any difficulties/ease for the insect to feed in vivo or on membranes.

h) Unfortunately, the feeding duration of insects in the different experimental groups was not assessed in this study. During the feeding assays, all groups were allowed access to the blood source for 90 min.

• Page 12, line 31 There is an error in “IIn vitro”.

i) The typo has been corrected.

Nothing to add regarding the references, figures, and graphs.

### Reviewer 3:

Reviewer Report (for Memórias do Instituto Oswaldo Cruz – MIOC)

Manuscript ID: MIOC-2026-0065 Title: Application of image analysis in the evaluation of blood feeding by Lutzomyia longipalpis using an artificial feeding system with synthetic membranes.This manuscript addresses an important methodological bottleneck in sand fly research: improving artificial feeding and implementing image-based, non-destructive blood-meal quantification. The comparison of membranes (chick skin vs hydrated collagen ± plasma gel) and the Fiji/ImageJ workflow are timely and potentially valuable for colony maintenance and experimental studies.

Response: We thank the reviewer for their valuable comments and suggestions.

Introduction:

The Introduction would benefit from being more focused and concise. At present, it feels somewhat long and includes some repeated background points (e.g., multiple statements about ethical/logistical limitations of in vivo feeding and the general importance of membrane choice and artificial feeding).

a) The Introduction has been revised to improve conciseness.

Methods:

Page 4 line 51: Explain the expression “closed colony” as follow: A closed breeding colony of Lu. longipalpis (originating from Teresina, Piauí, Brazil) has been maintained in the insectary of the Laboratory of Haematophagous Insect Physiology (ICB–UFMG) since 2009 under controlled conditions (25 ± 1 °C; 80–95% relative humidity).

b) As suggested by the reviewer, the term “closed colony” has been removed from the manuscript:

“The sand flies have been maintained as a closed colony at the insectary of the Laboratory of Haematophagous Insect Physiology (ICB–UFMG) since 2009, under controlled temperature (25 ± 1 °C) and relative humidity (80–95%).”

Page 5 Line 8: Format species name in italic.

c) We have corrected the formatting by italicizing *Mesocricetus auratus* throughout the manuscript.

Feeding assays: The groups acronyms are not self-explanatory and can make interpreting the results difficult. Suggest the authors to change the acronyms and include a sentence in the methodology: Groups were: IVH (in vivo hamster), CHK (chick skin membrane), COL+PG (hydrated collagen treated with plasma gel), COL (hydrated collagen untreated), and UNF (unfed control).

d) Following the reviewer’s suggestion, the abbreviations of the experimental groups have been revised throughout the manuscript.

Explain that the negative control “unfed” group was used to establish a criterion of abdominal area to classify the insects as engorged or unfed. The criterion uses the negative control mean + 2 SD threshold. Ensure it is described clearly as an analytic reference and that sample sizes used for thresholding.

e) Following the reviewer’s suggestion, the text concerning the negative control group (unfed) has been rewritten for improved clarity.

“A subset of females from the chick skin membrane group (17 females) exhibited atypical abdominal coloration. Because visual assessment alone could not reliably confirm blood ingestion, females with abdominal areas exceeding the unfed group (negative control, n=240) mean plus two standard deviations (0.62 mm) were considered engorged”.

Page 5 Line 35: Abbreviate the genus Mesocricetus, as it is already written in full on line 8.

f) The genus name Mesocricetus has been abbreviated to M. auratus where appropriate.

“Golden hamsters (Mesocricetus M. auratus; 40–60 days old, 100–150 g) were obtained…”

Page 5 Line 40: Change One to A group.

g) Following the reviewer’s suggestion, the sentence was corrected:

“Five experimental groups were tested: a group fed on a live host”

Page 5 Line 40: Include how many sand flies were used and was only females or males together. The number of insects used should be described in each group in the methodology.

h) The sample size (number of insects) for each experimental group was added, as well as the total number of males and females in the entire study:

“A total of 1300 female and 217 male sand flies, 5–7 days post-emergence, were used…”

Page 5 Line 54: The sentence of 12h starved is already explained in Feeding assay

i) The following sentence has been removed from the Materials and Methods section (sub-item ii, Artificial feeder):

“Flies were starved for 12 h prior to assays to enhance feeding avidity.”

Page 5 Line 57: Is there any ethics license for the human blood used?

j) The ethics approval number for the use of human blood has been added to the manuscript.

“Human blood usage followed the guidelines of the Research Ethics Committee of the Federal University of Minas Gerais (CEP/UFMG protocol 4.512.912).”

It would improve clarity if the Methods description of all feeding conditions followed the same internal structure within the “Feeding assays” section. The authors introduce the subsections “Live host” and “Artificial feeder,” but the subsequent description of membrane treatments (chick skin vs. collagen ± plasma gel) is presented as a separate block rather than as a continuation of the artificial-feeder workflow. As a result, readers must piece together which procedures correspond to each experimental group and how many sand flies were used per feeding condition. I recommend reorganizing this part of the Methods so that each feeding group is described in a parallel format (e.g., group definition → number of females/males per replicate → feeding duration/temperature → membrane preparation → post-feeding handling), or alternatively nesting “Membrane treatments” as a sub-subsection under “Artificial feeder.” This would make it much easier for readers to understand the experimental groups and the sample size used in each feeding procedure.

k) This section has been moved from the “Membrane treatments” subsection to the “Artificial feeder” subsection:

ii) Artificial feeder

“Blood meals were offered using a Hemotek system (Hemotek Ltd., UK) at 37 °C for 90 min. Human blood usage followed the guidelines of the Research Ethics Committee of the Federal University of Minas Gerais (CEP/UFMG protocol 4.512.912).”

For blood meals assays in artificial feeder were used three feeding membranes: (i) chick skin (positive control; CHK group, n = 235) (19); (ii) hydrated collagen membrane (Hemotek Feeding Membrane, Hemotek Ltd., UK) treated with plasma gel (COL+PG group, n = 240); and (iii) untreated hydrated collagen membrane (COL group, n = 225). Blood in the Hemotek reservoir was carefully homogenised halfway through the feeding period (~45 min). All experiments were performed under controlled insectary conditions (25 ± 1 °C; 80–95% RH).”

Page 6 Line 49: Avoid abbreviations that may overlap with widely used terms. “ABS” is commonly read as “absorbance,” whereas “RCS” is not intuitive or self-explanatory to readers. Use the suggestion below:

ABS (whole abdominal area) → A_abd (abdominal area) or AbdA (Abdominal Area)

RCS (red-coloured blood area) → A_blood (blood area) or RCS → BldA (Blood Area)

Include the following sentence in the section Image acquisition: Blood meal size was estimated by image analysis using two ROIs: abdominal area (A_abd) and blood/red area (A_blood).

l) Following the reviewer’s suggestion, the abbreviations have been changed throughout the manuscript.

Page 6 line 32 - The text states that dry vs hydrated collagen differed significantly but reports “t-test p>0.005” Please correct the p-value direction/threshold p<0.05.

m) The p-value has been corrected in the manuscript.

Page 7 – Cite figure 3 in the methods.

n) Indeed, Figure 3 was inadvertently omitted from the text. A citation to this figure has now been added:

“The abdominal contour was then delineated after conversion to 8-bit grayscale, threshold adjustment, and automatic boundary detection using the Wand (tracing) tool (Fig. 3A-B)”

Results:

Page 8, lines 41. “17 of 235 females (0.03%) were reclassified as engorged.” This percentage is mathematically incorrect (17/235 ≈ 7.2%). The authors must correct.

o) The reviewer is correct, and the percentage has been corrected accordingly.

“According to this criterion, 17 of 235 females (7.2 %) were reclassified as engorged, despite their pale abdominal content.”

Page 9, lines 47 In the Results, the correlation between ABS and RCS (R² = 0.85) is described and then referenced to “Fig. 5,” but Fig. 5 is the barplot of feeding percentages; the regression is shown in Fig. 7. Please correct the figure reference(s) to avoid reader confusion.

p) The text below has been corrected:

“Linear correlation analysis of females with both ROIs measured demonstrated a strong positive association between abdominal area and red-coloured region area (R² = 0.85) (Fig.7).”

Discussion

There are sections of justification and introduction in the Discussion that could be removed.

q) Several sections of the Discussion have been revised to improve the flow and clarity of the arguments.

Some paragraphs lack coherence and connection with each other.

r) Please see the response above.

On page 12, there is double space between paragraphs.

s) The formatting issue on page 12 has been corrected.

The sentence between lines 14 and 21 on page 13 would be better placed after the end of the first paragraph on page 12, as it would also help explain the importance of including females that fed only on plasma and few erythrocytes.

t) This part of the discussion has been entirely rewritten:

“The implementation of artificial feeding as an alternative to the use of live hosts for the maintenance of laboratory sand fly colonies must take into account that the colonization of these vectors remains restricted to a relatively small number of specialized laboratories (23). Although Lutzomyia longipalpis is one of the main experimental models used in leishmaniasis research, information regarding physiological changes and the cumulative effects of infection over successive gonotrophic cycles remains limited, largely due to the high mortality observed in females after the first oviposition (44). This scenario contrasts with that of mosquitoes, whose laboratory rearing has been refined for more than a century. Currently, numerous mosquito species can be maintained for many generations using artificial feeding systems and a variety of blood sources, including isolated blood components such as plasma supplemented with phagostimulants (45). Consequently, the effects of different dietary compositions on feeding behavior, reproduction, and overall biological performance have been extensively documented in mosquitoes (46-50).”

It is also necessary to include in the Discussion the rationale for adding plasma gel to the membrane.

u) We have clarified the rationale for using plasma gel in the Materials and Methods section. In addition, we have expanded the Discussion to compare our findings with those of Hošková et al. (2025), who used CBP (coagulated and dried blood plasma) as an olfactory stimulus.

Materials and Methods:

“Heparinised human blood (0.04 µL heparin/mL blood) collected by venipuncture into Vacutainer® tubes was used both for feeding and plasma gel preparation. Plasma gel was applied to the collagen membrane to provide an olfactory stimulus and enhance feeding attraction.

Discussion:

“Additionally, experiments using frog skin as a feeding membrane were unsuccessful. To enhance membrane attractiveness and improve feeding success, Hošková et al. (20) coated the outer surface of both chicken skin and synthetic membranes with coagulated blood plasma (CBP) before offering them to S. minuta and other species exhibiting low feeding rates. Although this treatment significantly improved feeding success in most species tested, no increase in blood-feeding rates was observed in Lu. longipalpis fed through a synthetic membrane.”

Page 12 line 31“IIn vitro feeding…” (extra “I”).

v) This mistake has been corrected

Decimal punctuation: Please standardize decimal separators in English text/tables/figures. The manuscript mixes “0.84” and “0,84” and “p<0,05.” Use “0.84” and “p<0.05” consistently.

w) Decimal notation has been checked and corrected throughout the manuscript.

Figure legends should have a Title (a short, informative title that captures the whole figure), Groups/conditions, description of each panel (A, B, C…), briefly methodology, definition of all experimental groups/conditions and expand any abbreviations used in the figure, symbols/annotations, sample size (n), replicates and statistics.

x) The figure legends have been reviewed and revised.

Figure 1 could be moved to Supplementary materials

y) We appreciate the reviewer’s comment on Figure 1. However, we prefer to retain Figure 1, as it illustrates an essential methodological step regarding the plasma gel aspect, which is crucial for the reader’s understanding of the study.

Figures 1–4 are illustrative; consider adding “representative images”

z) We thank the reviewer for this valuable suggestion. The term ‘representative images’ has been included in the captions for Figures 1–4

Figure 7 legend - should include key regression statistics (n, R², p-value, and ideally regression equation and CI).

aa) The legend of Figure 7 has been revised to improve clarity:

“Figure 7: Linear regression between abdominal region area (AbdA) and blood-fed region area (BldA) derived from image analysis of 669 fed Lutzomyia longipalpis females. The line equation is Y = 0.8320*X – 0.04417, with R²= 0.8531 and p < 0.0001. Dashed lines indicate the 95% confidence interval (0.8008 to 0.8633)”

References: include DOI

Ab) As requested by the reviewer, the DOIs have been added to the references.

# Reviewer report: REVIEWERS’ COMMENTS

## REVIEWER #1

The article has been revised in accordance with all the recommendations requested by the reviewers and is considered suitable for publication.

## REVIEWER #2

The article is more concise after the first round of revisions; therefore, it can be published.

## REVIEWER #3

No comments.
